# Oral Health Care for Individuals With Special Needs: Practices and Challenges Among General Practitioners in Saudi Arabia

**DOI:** 10.7759/cureus.75988

**Published:** 2024-12-19

**Authors:** Saja Alhaidar, Lojaen S Sendy, Laila A Alanzan, Abdulrahman Z Alshehri, Ahmed S Alduraywishi, Ameera Althaqeel, Yazeed A Alzamel, Albandari Aldaws

**Affiliations:** 1 Pediatric Dentistry, King Saud Medical City, Riyadh, SAU; 2 Dentistry, King Saud Medical City, Riyadh, SAU; 3 College of Dentistry, King Saud University, Riyadh, SAU; 4 Dentistry, Riyadh Elm University, Riyadh, SAU; 5 Dentistry, Princess Nourah Bint Abdulrahman University, Riyadh, SAU

**Keywords:** dental care access, dental training, disabilities and dental care, general practitioners, healthcare challenges, oral health barriers, oral health care, saudi arabia, special healthcare needs (shcn), special needs dentistry

## Abstract

Introduction: A vital component of public health that needs a lot of attention is oral health care for people with special needs. The phrase "special needs" describes a wide range of issues pertaining to behavior, development, health, and emotions that require specific medical and educational support. These individuals often present with complex oral health care needs that require specialized knowledge and skills. Despite their significant numbers, access to quality oral health care for this group remains a critical concern.

Objectives: The objective of this study was to evaluate the practices and challenges faced by general practitioners (GPs) in delivering oral health care to individuals with special needs.

Methods: The sample size of this study consisted of 380 GPs. Informed consent was obtained from the participants, and they were informed that their responses and personal information would be kept confidential. A cross-sectional survey in English was prepared using Google Forms. The survey comprising 19 questions was distributed among a sample drawn from various regions of Saudi Arabia. Participants were selected to ensure representation from different practice settings, including private clinics, government hospitals, and university clinics.

Result: A significant portion of participants (271, 71.3%) reported lacking formal training in dental care for individuals with special needs. Furthermore, 277 (72.9%) expressed low confidence in their ability to provide care for these patients. Additionally, 342 (90%) acknowledged that the challenges in treating patients with special needs significantly impact their ability to deliver effective care. Moreover, 326 (85.8%) indicated insufficient access to the necessary resources and support, pointing to critical gaps in the provision of dental services for individuals with special needs.

Conclusion: The findings of this study showed that GPs encounter significant challenges in treating individuals with special needs in daily clinical settings. These challenges contribute to insufficient training of GPs in the field, limited resources provided, and inadequate support. The majority of participants in this study believe that a lack of professional guidelines, trained support staff, and continuing education opportunities contribute significantly to the challenges they face in delivering oral health care to individuals with special needs.

## Introduction

A vital component of public health that needs a lot of attention is oral health care for people with special needs. The phrase "special needs" describes various issues pertaining to behavior, development, health, and emotions that require specific medical and educational support. Over a billion individuals, or 16% of the world's population, are thought to live with disabilities globally; many of them need specialized oral health care [1]. In Saudi Arabia, about 5.9% of the population lives with a disability [2]. These individuals often have complex oral health needs that require specialized skills and knowledge. However, despite their significant numbers, access to quality oral health care remains a pressing concern for this group. 

People with special healthcare needs (SHCN) sometimes face complicated obstacles when seeking adequate dental care services. This category consists of individuals with disabilities necessitating medical care. Oral health plays a role in health and presents additional challenges for individuals with SHCN due to difficulties in maintaining regular oral hygiene routines, behavioral challenges, and the limited availability of dental professionals trained to address their needs [3].

Unfortunately, oral health issues are more prevalent among patients with SHCN compared to the general population, with studies consistently reporting higher rates of dental caries, periodontal disease, and other complications [3]. Despite the clear need for specialized care, barriers such as financial limitations, lack of insurance coverage, transportation challenges, and insufficient provider knowledge and training often prevent patients with SHCN from receiving the necessary care [4].

Patients with special needs often require polymedication due to their complex health conditions and comorbidities, which can significantly impact their oral health. For instance, individuals with epilepsy who take anticonvulsant drugs may develop gingival hyperplasia, increasing the risk of periodontal disease and making it more challenging to maintain good dental hygiene [5]. Additionally, children with cerebral palsy frequently experience gastroesophageal reflux, leading to enamel erosion and subsequent tooth decay [6].

Many medications prescribed for chronic conditions can cause xerostomia (dry mouth), gingival overgrowth, and altered taste sensation, all of which heighten the risk of dental caries, periodontal disease, and oral infections [5]. Furthermore, drug interactions can complicate dental treatments, making it essential for dental professionals to have a comprehensive understanding of their patients' medication regimens [7]. Barriers to accessing specialized dental care, such as a lack of trained professionals and inadequate facilities, exacerbate these oral health challenges in populations with special needs [4,6]. Educational interventions aimed at improving the knowledge and attitudes of dental practitioners toward special needs care have been shown to enhance the quality of oral health services provided, potentially reducing the negative impact of polymedication on oral health [8].

A multimodal strategy that incorporates specialist treatments catered to this population's demands in addition to normal dental care practices is necessary for the effective management of these issues. It has been demonstrated that methods including desensitization, sedation, and the employment of a multidisciplinary team that includes behavioral specialists and occupational therapists improve patient results [7]. To further enhance the standard of care, dental professionals must get ongoing education and training in managing patients with special needs [9].

General practitioners (GPs) play a crucial role in providing oral healthcare to individuals with SHCN. However, there is limited research on the challenges they face in delivering this care, particularly in Saudi Arabia. Studies consistently indicate that GPs often lack the necessary knowledge and skills to properly address the unique dental needs of patients with SHCN [8]. Additionally, caregivers frequently report that GPs are insufficiently trained and lack access to critical resources, such as specialized equipment and trained staff, which compromises the quality of care [10].

Several common challenges include limited specialized knowledge, insufficient training, and a lack of essential resources. Healthcare facilities often fail to prioritize investments in equipment and infrastructure tailored for patients with special needs, further hindering effective care [11]. Moreover, individuals with SHCN encounter significant barriers to accessing dental services, including transportation difficulties and communication issues, which often lead to delayed or missed appointments, worsening their oral health problems [12].

This research focuses on examining how practitioners (GPs) face challenges and facilitate providing care for individuals with special needs. By analyzing existing methods, identifying barriers, and evaluating the availability of training and resources, this research aims to provide valuable insights that will inform the development of targeted interventions and policies. Ultimately, the goal is to enhance healthcare services, improve accessibility, and ensure the inclusion and well-being of all citizens, particularly those with special healthcare needs, thereby contributing to the overall improvement of oral healthcare standards.

## Materials and methods

This study employed a cross-sectional survey design to collect data from GPs across Saudi Arabia. The primary objective was to assess the practices and challenges faced by GPs in providing oral health care to individuals with special needs. The survey, developed in English using Google Forms, consisted of structured questions to gather relevant data. A total of 380 GPs participated in the study. A stratified random sampling method was employed to select participants. Saudi Arabia was stratified into five major regions: central, eastern, western, northern, and southern. Within each region, GPs were randomly selected to participate, ensuring representation across diverse geographic and professional settings. The sample included practitioners from private clinics, government hospitals, and university clinics. To minimize selection bias, randomization within each stratum was conducted, and efforts were made to recruit a diverse pool of participants. Participation was voluntary, and multiple reminders were sent to ensure an adequate response rate.

The inclusion criteria were GPs actively practicing in Saudi Arabia, practitioners who consented to participate in the study, and participants with access to digital tools to complete the electronic survey. The exclusion criteria were dentists not practicing in Saudi Arabia, dentists not practicing as GPs, and participants who provided incomplete survey responses.

The questionnaire consisted of 19 questions and three main sections. The first five questions were to gather basic demographic data, including age, gender, years of practice, practice setting, and geographic location. Followed by three questions to assess the respondents' knowledge and training related to the treatment of individuals with special needs. These included self-assessment of their knowledge of special needs dentistry; the extent of formal education or training received on treating special needs patients; perception of the adequacy of their training and readiness to treat patients with special needs. The last eleven questions were focused on the specific practices and challenges faced by GPs when treating patients with special needs. These included the frequency of treating patients with special needs; types of dental procedures commonly performed on special needs patients; use of specialized equipment and techniques (e.g., sedation, general anesthesia); common challenges encountered such as communication barriers, lack of specialized equipment, and difficulty in managing patient behavior; perception of institutional and systemic support for treating special needs patients (e.g., availability of resources, referral systems); open-ended questions to provide additional insights into specific challenges and suggestions for improvement.

Ethical approval

The study adhered to ethical guidelines and received an exemption letter from the Institutional Review Board (IRB) under Registration Number KACST, KSA: H-01-R-053, registered with the U.S. Department of HHS IORG #: IORG0010374. Informed consent was obtained from all participants, who were assured of the confidentiality of their responses and personal information.

Data analysis

The data collected through the electronic questionnaire were entered and analyzed using IBM SPSS Statistics for Windows, Version 25.0 (Released 2017; IBM Corp., Armonk, New York, United States). Descriptive statistics were generated for all study variables, with the results presented as frequency tables, percentages, and figures. For questions with multiple-choice responses, each option was analyzed separately to determine the distribution of responses across the sample, ensuring detailed representation of the data. Inferential statistics were applied to identify significant associations, with the chi-square test used for analysis, as all study variables were nominal or ordinal. This approach provided robust insights into the patterns and relationships within the dataset.

## Results

Participant’s demographic information

A total of 380 GPs participated in this study, with a gender distribution of 214 (56.3%) males and 166 (43.7%) females (Table 1). The majority of participants (274, 72.1%) graduated from government colleges, while 106 (27.9%) were from private colleges (Table 2). Regarding the region of practice, 207 (54.5%) were based in the central region, 78 (20.5%) in the western region, 47 (12.4%) in the eastern region, 25 (6.6%) in the northern region, and 23 (6.1%) in the southern region (Table 3). In terms of years of practice, 224 (58.9%) of participants had less than five years of experience, 117 (30.8%) had 5-10 years, 32 (8.4%) had 11-15 years, and 7 (1.8%) had 16-20 years (Table 4). The distribution of practice settings revealed that 233 (61.3%) were in private clinics, 116 (30.5%) in government hospitals, and 31 (8.2%) in university clinics (Table 5).

**Table 1:** Gender of participants.

Gender	Frequency	Percent
Male	214	56.3
Female	166	43.7
Total	380	100.0

**Table 2:** Type of college.

Type of college	Frequency	Percent
Government	274	72.1
Private	106	27.9
Total	380	100.0

**Table 3:** Practice region.

Practice region	Frequency	Percent
Central	207	54.5
Eastern	47	12.4
Western	78	20.5
Northern	25	6.6
Southern	23	6.1
Total	380	100.0

**Table 4:** Years of practicing as GP. GP: general practitioner.

Years of practice	Frequency	Percent
Less than 5 years	224	58.9
5-10 years	117	30.8
11-15 years	32	8.4
16-20 years	7	1.8
Total	380	100.0

**Table 5:** Type of practice setting.

Type of practice setting	Frequency	Percent
Private clinic	233	61.3
Government hospital	116	30.5
University clinic	31	8.2

Knowledge and training

Regarding knowledge and training, as shown in Table 6, the majority of participants (271, 71.3%) do not receive any formal training in providing dental care for individuals with special needs. However, 109 (28.7%) received training. Of those, 60 (55%) participants received formal training in providing dental care for individuals with special needs as a clinical attachment, 26 (23.9%) from undergraduate dental school, five (4.6%) from the postgraduate program, three (2.8%) in terms of continuing education courses, and two (1.8%) as workshops or seminars. Additionally, 13 (11.9%) of the participants did not provide information on their training status (Table 7).

**Table 6:** Training.

Receiving training	Frequency	Percent
Yes	109	28.7
No	271	71.3
Total	380	100.0

**Table 7:** Training providers (n=109, missing cases 11.9%).

Training providers	Frequency	Percent
Undergraduate dental school	26	23.9
Postgraduate program	5	4.6
Continuing education courses	3	2.8
Workshops or seminars	2	1.8
Clinical attachment	60	55.0
Total	96	100.0

As shown in Table 8, the confidence level of participants in providing dental care to patients with special needs revealed that most participants (277, 72.9%) are not confident. However, 41 (10.8%) reported they were confident in providing dental care to patients with special needs. Additionally, 62 (16.3%) of participants did not give an exact response about this issue, i.e., they were neutral.

**Table 8:** Confidence in providing dental care to patients with special needs.

Competency level	Frequency	Percent
Not confident at all	61	16.1
Not very confident	216	56.8
Neutral	62	16.3
Confident	38	10.0
Very confident	3	0.8
Total	96	100.0

Inferential statistics

1. Did participants with training report higher confidence levels?

The data in Table 9 highlight a significant association between receiving formal training and increased confidence levels in providing dental care to individuals with special needs. Among participants who did not receive formal training, 54 (88.5%) reported low confidence levels ("Not confident at all"), while only seven (11.5%) of formally trained participants reported similarly low confidence. Conversely, confidence was markedly higher among those who received formal training, with 34 (89.5%) reporting they were "Confident" and one (33.3%) reporting they were "Very confident."

**Table 9:** Training and confidence levels.

How would you rate your confidence in providing dental care to patients with special needs?		Receiving formal training		Total
		Yes	No	
Not confident at all	7		54	61
	11.5%		88.5%	100.0%
Not very confident	40		176	216
	18.5%		81.5%	100.0%
Neutral	27		35	62
	43.5%		56.5%	100.0%
Confident	34		4	38
	89.5%		10.5%	100.0%
Very confident	1		2	3
	33.3%		66.7%	100.0%
Total		109	271	380
		28.7%	71.3%	100.0%

Statistical analysis confirms this relationship, with Pearson chi-square statistics yielding χ²=89.440, p=0.000, indicating a statistically significant association between formal training and confidence levels. Furthermore, the symmetric measures revealed a moderate positive correlation (r=0.447), underscoring the importance of formal training in enhancing confidence levels among participants.

2. Was the type or source of training (e.g., clinical attachment, continuing education) associated with better competence or confidence?

The results presented in Table 10 indicate that the type or source of formal training did not show a statistically significant association with competence or confidence levels in providing dental care to patients with special needs (Pearson chi-square statistics: χ²=21.740, p=0.155). Additionally, the symmetric measures revealed a weak relationship between the source of formal training and confidence levels (correlation coefficient, r=0.381).

**Table 10:** Type or source of training and competence or confidence.

Confidence in providing dental care to patients with special needs	Type or source of training					Total
	Undergraduate dental school	Postgraduate program	Continuing education courses	Workshops or seminars	Clinical attachment	
Not confident at all	3	0	0	0	3	6
	50.0%	0.0%	0.0%	0.0%	50.0%	100.0%
Not very confident	9	1	1	0	21	32
	28.1%	3.1%	3.1%	0.0%	65.6%	100.0%
Neutral	11	1	1	0	11	24
	45.8%	4.2%	4.2%	0.0%	45.8%	100.0%
Confident	3	3	1	2	24	33
	9.1%	9.1%	3.0%	6.1%	72.7%	100.0%
Very confident	0	0	0	0	1	1
	0.0%	0.0%	0.0%	0.0%	100.0%	100.0%
Total	26	5	3	2	60	96
	27.1%	5.2%	3.1%	2.1%	62.5%	100.0%

Clinical practices

The participants were tested regarding how often they treat patients with special needs in their practice. The results revealed that more than half of participants, 220 (57.9%), had monthly experience treating patients with special needs. However, 117 (30.8%) of participants reported rarely, and 34 (8.9%) reported weekly. Only two (0.5%) participants reported daily practice (Table 11).

**Table 11:** Frequency of treating patients with special needs.

Practice	Frequency	Percent
Daily	2	0.5
Weekly	34	8.9
Monthly	220	57.9
Rarely	117	30.8
Never	7	1.8
Total	380	100.0

As shown in Table 12, Down syndrome was the most common special need encountered most frequently by participants (238, 62.6%), followed by mental disability (207, 54.5%) and physical and motor impairment (168, 44.2%). On the other hand, speech and communication disorders and autism got the same percentage (154, 40.5%), followed by behavioral and emotional disorders (137, 36.1%) and learning difficulties (126, 33.2%). However, visual impairment was the least special need encountered by 23 participants (6.1%).

**Table 12:** Types of special needs encountered most frequently.

Responses	No	Percent
Visual impairment	23	6.1
Hearing impairment	0	0
Mental disability	207	54.5
Physical and motor impairment	168	44.2
Learning difficulties	126	33.2
Speech and communication disorders	154	40.5
Behavioral and emotional disorders	137	36.1
Autism	154	40.5
Down syndrome	238	62.6

Table 13 shows the modifications made to accommodate patients with special needs. Additional support staff (210, 55.3%) came first, followed by extended appointment times (199, 52.4%), sedation or general anesthesia (154, 40.5%), customized communication techniques (140, 36.8%), and specialized equipment (126, 33.2%).

**Table 13:** Modifications made to accommodate patients with special needs.

Responses	No	Percent
Extended appointment times	199	52.4
Sedation or general anesthesia	154	40.5
Specialized equipment	126	33.2
Additional support staff	210	55.3
Customized communication techniques	140	36.8

Challenges and barriers when treating patients with special needs

As shown in Table 14, time constraints were the most challenging faced by GPs when treating patient with special needs (227, 20.3%), followed by lack of training (221, 19.8%), inadequate support staff (189, 16.9%), lack of specialized equipment (167, 14.9%), behavioral management issues (158, 14.1%), and communication barriers (156, 14%). 

**Table 14:** Main challenges faced by participants when treating patients with special needs.

Responses	No	Percent
Lack of training	221	19.8
Lack of specialized equipment	167	14.9
Communication barriers	156	14.0
Behavioral management issues	158	14.1
Time constraints	227	20.3
Inadequate support staff	189	16.9

The results in Table 15 revealed that challenges faced by participants when treating patients with special needs were very important and had an impact on their ability to provide care to patients with special needs, as reported by 228 participants (60%). However, it can be concluded that the majority of participants, 342 (90%), believe that challenges faced when treating patients with special needs are important and have an impact on the ability to provide care to patients with special needs.

**Table 15:** Effects of challenges in participant’s ability to provide care to patients with special needs.

Responses	No	Percent
Not significant	3	0.8
Minor	7	1.8
Moderate	28	7.4
Significant	114	30.0
Very significant	228	60.0

Resources and support

Resources and support were investigated in terms of having access to adequate resources and support to provide care to patients with special needs, in addition to investigating the lack of some resources as perceived by study participants. The results revealed that the majority (326, 85.8%) of the study sample reported having no access to adequate resources and support, while 54 (14.2%) reported having access to resources and support (Table 16).

**Table 16:** Access to adequate resources and support.

Responses	No	Percent
Yes	54	14.2
No	326	85.8

As shown in Table 17, professional guidelines are the most common resource lacking, as reported by study participants (228, 31.3%), followed by trained support staff (161, 22.1%), continuing education opportunities (151, 20.7%), specialized equipment (108, 14.8%), and financial support (81, 11.1%).

**Table 17:** Lacking resources as reported by study participants.

Responses	No	Percent
Specialized equipment	108	14.8
Trained support staff	161	22.1
Continuing education opportunities	151	20.7
Financial support	81	11.1
Professional guidelines	228	31.3

Recommendations

The study participants provided valuable insights into the additional training and resources they believe would be most beneficial in improving care for patients with special needs. Below are the key recommendations based on their feedback:

Additional Training and Resources Needed

The participants highlighted the following as essential areas for improvement in providing oral health care to individuals with special needs (Table 18).

**Table 18:** Additional training or resources needed.

Responses	No	Percent
Hands-on workshops	122	10.5
Online courses	123	10.5
Conferences and seminars	252	21.6
Access to specialized equipment	206	17.7
Hiring of trained support staff	196	16.8
Development of professional guidelines	268	23.0

Participant Recommendations for Improving Oral Health Care for Individuals With Special Needs in Saudi Arabia

Participants recommended several improvements in oral health care for individuals with special needs in Saudi Arabia. Key suggestions included making dental clinics more accessible by equipping them with facilities for those with physical disabilities. Expanding residency programs for family and special needs dentistry, in collaboration with top dental schools abroad, was also highlighted. Participants advocated for more training opportunities at the undergraduate level through seminars and specialized postgraduate training. Additionally, they emphasized the importance of periodic training and services for GPs, along with providing guidance and support to caregivers and families.

Positive Experiences With Treating Patients With Special Needs

Participants shared their positive experiences treating patients with special needs, emphasizing the rewards of such care. They highlighted the importance of building trust, creating a calming environment, and using a patient-centered approach that involves patience, clear communication, and positive reinforcement. Many participants found personal fulfillment in helping these patients, especially when offering home treatment services, which increased patient satisfaction. Treating patients with special needs provided valuable learning opportunities, allowing practitioners to adapt treatment plans. For broader improvements, participants suggested continuous learning for dental professionals, establishing specialized clinics, promoting home dental services, and optimizing resources in both government and private sectors.

## Discussion

This discussion explores the challenges faced by GPs in Saudi Arabia when caring for patients with SHCN. It contrasts these results with those of other comparable research and draws attention to key factors such as inadequate support staff, time limits, communication obstacles, behavioral management problems, and inadequate training. The study also addresses specific conditions, including Down syndrome and autism, as well as disabilities such as hearing, vision, mental, physical, motor, learning, and speech issues.

Lack of training and specialized equipment

The study's result, which is consistent with both domestic and foreign research, was that 271 (71.3%) GPs lacked formal training to oversee patients with SHCN. A significant number of Saudi dental care providers felt underprepared due to a lack of readily available training programs, echoing the critical need for specialized training in dental schools to adequately prepare practitioners for handling patients with SHCN [13]. Research has shown that dental professionals treating patients with Down syndrome in Saudi Arabia typically lacked adequate training to meet their complicated care demands, further highlighting this educational gap [14]. The lack of formal education and training in treating patients with SHCN is a prevalent challenge, especially in underdeveloped countries like Saudi Arabia, where training resources are more restricted, underscoring the worldwide character of this issue [15]. These results are consistent with findings from various studies conducted internationally in other areas. For example, many Saudi dentists recognized the need for more formal education on how to manage patients who are mentally and physically challenged [16]. This sentiment aligns with findings that one of the biggest obstacles to providing appropriate care for people with developmental and intellectual disabilities is a lack of specialized education and training [17]. Additionally, the insufficient inclusion of SHCN patient care in dental curricula worldwide limits practitioners' ability to manage these patients effectively [18,19].

Furthermore, 126 (33.2%) of GPs in the study mentioned the problem of not having the necessary specialized equipment, which is a persistent obstacle worldwide. The standard of care provided to patients with SHCN is restricted by the absence of specialist dental instruments in general dental offices [20]. These results align with international studies; resource constraints confront many dental practitioners globally, underscoring the possibility of subpar dental care for patients with SHCN without the necessary instruments [21]. Additionally, Saudi Arabian practitioners often faced insufficient access to the tools they needed, which had an immediate negative impact on the way patients with SHCN were treated [10]. The lack of specialized equipment can hinder the delivery of adequate care and prevent practitioners from effectively addressing the unique needs of these patients. Such limitations not only affect treatment outcomes but may also lead to increased anxiety and discomfort for both patients and providers. Addressing these equipment shortages is crucial for improving the overall quality of dental care for individuals with special healthcare needs. Additionally, ensuring that dental practices are adequately equipped can enhance the practitioners' confidence when treating this vulnerable population. Ultimately, investing in the necessary tools and resources is vital for advancing dental care standards and ensuring that all patients receive the quality care they deserve. 

The scarcity of specialty dental chairs, anesthetic instruments, and diagnostic tools in dentistry practices has made it more difficult to treat patients with physical disabilities [22]. Furthermore, GPs frequently struggle to provide appropriate care due to a lack of contemporary technology and facilities designed for patients with SHCN [23]. These equipment shortages restrict treatment options and negatively impact the overall quality of care. The need for investments in technology and equipment specifically designed to meet the demands of patients with complicated healthcare needs has been emphasized in the global discourse on improving dental care accessibility and effectiveness [24]. Addressing these deficiencies is essential not only for enhancing treatment outcomes but also for fostering an environment where practitioners feel equipped to manage the complexities of care for individuals with special needs. Furthermore, upgrading dental practices with modern equipment can improve the patient experience, reducing anxiety and increasing comfort during treatment. As the healthcare landscape evolves, ensuring that dental facilities are adequately equipped will be critical for meeting the needs of diverse patient populations. Ultimately, prioritizing these investments will contribute significantly to advancing the standards of care for patients with SHCN. Dental clinics frequently lack the resources to provide proper care for children with SHCN, particularly in urban locations like Jeddah. This highlights a general need for both policy and infrastructural reforms within the healthcare system. The shortage of resources not only limits the quality of care that practitioners can deliver but also creates significant barriers to accessing essential services for vulnerable populations. Moreover, a major obstacle to providing care for autistic patients has been identified as a lack of resources, which prevents practitioners from implementing effective behavior management strategies due to insufficient tools and support [25]. These resource constraints underscore the necessity for targeted investments in both physical infrastructure and human resources to better support healthcare providers in managing patients with special needs. Policymakers must recognize the urgent need for reforms that facilitate the allocation of adequate resources and support systems, ensuring that healthcare facilities are equipped to meet the complex demands of patients with SHCN. Addressing these challenges will be crucial in improving the overall quality of dental care and ensuring that practitioners have the necessary tools to implement effective treatment and management strategies. Furthermore, a comprehensive approach that includes training and development for practitioners will be essential to bridge the gap between available resources and the needs of patients with SHCN. By prioritizing these areas, healthcare systems can move toward more inclusive and effective care for all individuals, regardless of their unique challenges. 

Communication barriers and behavioral management issues

When working with patients who have SHCN, GPs reported that one of the most significant challenges they faced was communication problems. This issue became particularly apparent when treating patients with Down syndrome, autism, or other speech and communication difficulties. It was noted that dentists frequently struggled to communicate effectively with patients who had learning challenges, which impeded their ability to deliver adequate treatment [13]. The importance of effective communication in enhancing access to care for children with SHCN has been emphasized in recent studies, further highlighting the necessity of addressing these barriers in clinical practice [25]. Similar communication difficulties have also been observed among dentists in various regions, indicating that this is a widespread concern affecting practitioners on a global scale [10]. Behavioral management concerns represent another significant worry among GPs. In this study, 277 GPs (72.9%) notably reported lacking confidence in managing the behavior of patients with SHCN effectively. This finding aligns with previous research indicating that many dentists felt unprepared to treat patients who are mentally and physically challenged, reflecting a broader trend of apprehension within the dental community [17]. The lack of confidence in managing behavioral issues is a critical barrier that needs to be addressed to improve the quality of care for these patients. Additional studies have corroborated these behavioral management challenges, underscoring the need for enhanced training and support for practitioners in this area [26]. Internationally, similar behavioral management issues have been documented, emphasizing that the challenges faced by GPs in Saudi Arabia are not unique. Calls for improved behavioral management training have been made across different countries, highlighting a common need for dental education programs to incorporate strategies for effectively managing patients with SHCN [27]. Such training could equip practitioners with the necessary skills and knowledge to navigate the complexities of treating this patient population, ultimately leading to better outcomes. Addressing communication and behavioral management challenges is crucial for ensuring that GPs are prepared to meet the needs of patients with SHCN effectively. By investing in targeted training and resources, the dental community can work towards overcoming these obstacles, ultimately enhancing the overall quality of care for individuals with special healthcare needs. 

Time constraints and inadequate support staff

Two of the most significant issues identified in the study were time constraints and a shortage of adequate support staff. Nearly 210 (55.3%) of practitioners reported that they required more support personnel when treating patients with SHCN. This sentiment aligns with findings from recent studies, indicating that caregivers of patients with SHCN in Saudi Arabia frequently view the lack of qualified staff as a major barrier to accessing appropriate care [21]. The impact of inadequate staffing is a pressing concern, as it not only affects the availability of necessary support for practitioners but also compromises the overall quality of care provided to these vulnerable patients. Globally, similar challenges related to staffing and time constraints have been observed, underscoring that this issue is not confined to Saudi Arabia. Studies have consistently shown that inadequate staffing levels and time limitations significantly hinder the ability of healthcare providers to deliver optimal care to patients with SHCN [14]. This international perspective highlights the need for systemic changes to address the workforce challenges faced by practitioners in various healthcare settings. By recognizing that these issues are widespread, healthcare authorities can work towards developing more effective staffing models and support systems. In the survey, 199 participants (52.4%) indicated that longer consultation times were necessary to treat patients with SHCN appropriately, particularly those with physical or motor limitations. This finding emphasizes the need for healthcare providers to allocate sufficient time for consultations to address the complex needs of these patients effectively. The challenges associated with time constraints become even more pronounced when treating patients who require sedation or specialized equipment, as additional considerations must be considered during their care [28]. Moreover, the issue of time restrictions faced by GPs when treating patients with SHCN has been documented in other studies, revealing a consistent pattern of difficulty in managing the demands of these cases. It is crucial for healthcare systems to acknowledge and address these time constraints to enhance the quality of care for patients with SHCN. By ensuring that practitioners have adequate time and resources, the potential for improved patient outcomes increases significantly. Addressing the dual challenges of time constraints and insufficient support staff is essential for enhancing the quality of care for patients with SHCN. This can be achieved by implementing policies that promote better staffing levels and by fostering an environment where practitioners are encouraged to take the necessary time to address the unique needs of each patient. Through these efforts, the healthcare system can make strides toward providing more comprehensive and effective care for individuals with special healthcare needs [24]. 

Sedation, general anesthesia, and behavioral management

In contrast to specialized centers and practices in other countries, GPs in this study reported using general anesthesia or sedation in 154 cases (40.5%) when treating patients with SHCN. This utilization rate highlights a significant difference in practice patterns compared to specialized facilities, where trained personnel and appropriate resources are more readily available. In Saudi Arabia, the rate of sedation usage is notably higher in specialist dental institutions, where staff members have received training in sedation protocols, allowing for safer and more effective administration of these techniques [29]. Such specialized training equips practitioners with the knowledge and skills necessary to manage patients with complex needs, thereby facilitating better overall care. Research indicates that sedation is often essential for managing patients with SHCN, particularly those who experience significant behavioral issues. The calming effects of sedation can alleviate anxiety and make dental procedures more tolerable for patients, resulting in a more positive experience for both patients and practitioners [10]. However, the lower utilization rate of sedation in general dentistry clinics may be attributed to several factors, including limited access to qualified anesthetists or sedation specialists, which presents a substantial barrier for GPs attempting to provide adequate care for their patients [30]. The challenges faced by GPs are compounded by the lack of specialized training in sedation techniques, which has been shown to significantly impact the quality of care delivered to patients with SHCN [31]. Many GPs may lack confidence in safely administering sedation, especially when treating patients with complex medical or behavioral conditions. This lack of training can lead to increased reliance on referral to specialized facilities, where practitioners are more adept at managing sedation protocols and addressing patient needs effectively. Furthermore, the logistical challenges associated with providing sedation in a general practice setting can be prohibitive. Implementing sedation protocols requires adequate infrastructure, including recovery spaces and monitoring equipment, which are often lacking in general dental practices [18]. As a result, the use of general anesthesia or deep sedation is frequently confined to hospital settings or specialized dental centers, where these resources are more readily available. This limitation underscores the need for improved access to training and resources for GPs to enhance their capability to provide sedation safely and effectively. Despite the potential benefits of sedation, GPs often underutilize general anesthesia in their practices due to training deficiencies and regulatory constraints [32]. This presents a significant obstacle for practitioners attempting to provide care for patients with severe behavioral or cognitive impairments, as these patients may require sedation to facilitate necessary dental treatments. Consequently, it is crucial for the healthcare system to address these barriers and ensure that GPs have the training and resources needed to administer sedation when appropriate. Moreover, there is a pressing need for additional training in behavioral management strategies within general practice. Non-pharmacological behavioral management approaches should be prioritized, as they offer valuable alternatives to sedation and can be effective in managing patient anxiety [17]. However, without sufficient training in these techniques, GPs may struggle to implement them effectively, leading to an increased reliance on sedation in specialized facilities. This reliance can hinder the ability of GPs to provide comprehensive care for patients with SHCN and limits their potential to develop effective strategies for managing challenging behaviors. Overall, enhancing the training and resources available to GPs is essential for improving the quality of care provided to patients with special healthcare needs. By addressing the barriers related to sedation, general anesthesia, and behavioral management, healthcare authorities can facilitate better outcomes for patients with SHCN and support GPs in their efforts to deliver effective and compassionate care. 

Treating patients with specific disabilities

The study found that GPs most frequently treated patients with Down syndrome and mental health issues. This pattern is consistent with what has been observed in Saudi Arabia, where patients with Down syndrome often require frequent dental care. The high rate of caries and oral hygiene difficulties in this community has been noted [26], as children with Down syndrome often have unique oral health demands due to factors such as hypotonia, bruxism, and delayed tooth eruption [28]. For this reason, routine dental visits are essential to their care [17]. Autistic patients present additional challenges due to sensory sensitivity and communication difficulties. Specialized management strategies such as desensitization and visual support have been recommended to improve patient compliance [10]. Challenges such as behavioral issues and dental anxiety frequently hinder appropriate treatment for autistic children, and practitioners often feel unprepared to manage these patients due to a lack of specific training in behavioral management strategies [13]. These difficulties extend to patients with other physical or mental health conditions. GPs often feel unconfident in managing these behavioral issues [20]. Patients with visual, auditory, or motor impairments are treated less frequently by GPs, who cite a lack of experience and confidence as key obstacles. Inadequate training in these areas is a common issue [17], and international research has shown that insufficient exposure to patients with disabilities during training contributes to practitioners' low confidence levels [16]. In addition to communication challenges, treating patients with learning disabilities is particularly difficult for dental practitioners [23]. A lack of knowledge about these conditions presents a substantial barrier to care. International studies also confirm these patterns, highlighting that practitioners in countries with underdeveloped training systems struggle to provide effective care for patients with SHCN [33]. Specialized care is often required for patients with Down syndrome due to the severity of their oral health problems. However, patients with other disabilities, including motor or sensory impairments, often receive less attention due to practitioner knowledge gaps [14]. This reflects a broader trend, where a lack of specialized training hinders the ability to provide comprehensive care for these populations. The need for specialized training in handling behavioral issues and sensory sensitivity is particularly crucial for practitioners treating patients with autism and other sensory impairments [15]. Continuous education and hands-on workshops have been suggested as effective ways to build practitioner confidence in managing various disabilities. Simulation-based training has also been proposed to bridge the gap between theoretical knowledge and practical application, providing GPs with the opportunity to handle complex cases in a controlled environment before transitioning to real-world situations [26]. Strengthening training programs and incorporating them into dental education is a common recommendation. By enhancing these programs, aspiring dentists can gain the necessary skills and confidence to treat patients with various disabilities [18]. This viewpoint is further supported by calls for the inclusion of disability-specific courses in dental school curricula worldwide, ensuring that future dentists are well prepared to manage the challenges posed by patients with special healthcare needs [33].

Recommendation for improvements to overcome the difficulties they encounter, survey participants suggested a variety of approaches, such as in-person workshops, official training programs, and online courses [13]. These answers are consistent with research findings that emphasize the need for ongoing education in dental treatment for patients with special healthcare needs (SHCNs) [17]. To enhance care for patients with SHCN in Saudi Arabia, dedicated training programs need to be established and maintained. This entails providing chances for practitioners to continue their professional development as well as incorporating such training into dentistry education at the undergraduate level [10]. Additionally, the creation of specially designed courses that address a range of disabilities is necessary, stressing that treating patients with SHCN effectively requires an awareness of behavioral management strategies [33]. Similarly, it was suggested that official training programs should be required to boost dentists' confidence in handling SHCN situations. Research from other countries also recommended including disability-specific training in the dentistry curriculum to ensure practitioners have a wider range of expertise, which could improve care [20]. Enhancing access to care requires structural reforms, such as the adoption of national guidelines. Creating strong national standards would ensure dental practices are consistent across various healthcare environments. Written guidelines and structured referral channels could also improve the standard of care and reduce disparities in the services received by patients with SHCN [23]. National actions must be implemented to ensure that dental practitioners obtain continuous professional development about SHCN. This includes interactive seminars and virtual classes that can provide real-world experience in managing challenging issues. The development of flexible, remote learning modules is also important to enable the acquisition of essential skills without overwhelming busy practitioners [16]. The importance of ongoing professional development through online courses and interactive seminars, as well as the creation of adaptable, remote learning modules, was also highlighted. These efforts would help bridge knowledge gaps and ensure practitioners have the skills needed to provide effective care to patients with SHCN while also addressing the logistical challenges faced by those with demanding schedules [25].

Study limitations

This study has a noteworthy limitation that merits consideration. The use of an online survey platform may have excluded participants with limited access to digital tools, thereby affecting the generalizability of the results. This should be considered when interpreting the study's results and formulating strategies for improvement.

## Conclusions

The oral healthcare needs of individuals with special needs are often complex and require specialized knowledge and skills. GPs encounter significant challenges in treating individuals with special needs in daily clinical settings. These challenges are attributed to insufficient training of GPs in the field, limited resources provided, and inadequate support. Most participants in this study believe that the lack of professional guidelines, trained support staff, and continuing education opportunities significantly contribute to the challenges they face in delivering oral healthcare to individuals with special needs. Furthermore, the role of caretakers is crucial, as their awareness of the potential oral health consequences of neglecting regular dental visits can significantly impact patient outcomes. The study emphasizes the importance of multidisciplinary approaches to improving oral health care for individuals with special needs by providing training programs for GPs, establishing guidelines, conducting educational campaigns for caretakers, and continuing education programs as key solutions.
